# Expression of somatostatin receptor subtypes in human thyroid tumors: the immunohistochemical and molecular biology (RT-PCR) investigation

**DOI:** 10.1186/1756-6614-2-1

**Published:** 2009-01-27

**Authors:** Hanna Pisarek, Tomasz Stępień, Robert Kubiak, Edyta Borkowska, Marek Pawlikowski

**Affiliations:** 1 Department of Neuroendocrinology, Medical University, Łódź, Poland; 2 Department of General and Endocrine Surgery, Medical University, Łódź, Poland; 3 Department of Pathology of Tumors, Medical University, Łódź, Poland; 4 Department of Medical Genetics, Medical University, Łódź, Poland

## Abstract

Human endocrine tumors often express the somatostatin receptors SSTR 1–5 with different intensity. It has been widely investigated their distribution in pituitary adenomas, brain tumors, adrenal tumors and neuroendocrine tumors in gastrointestinal tract (NET). Some of studies also concern the expression of SSTRs in thyroid tumors but they are mainly limited to parafollicular C cells – derived medullary thyroid carcinomas (MTC). Results of SSTR 1–5 detection in other thyroid pathologies like follicular adenomas and papillary cancers are still scarce and often controversial, depending of investigation method used. The aim of this study was to report the presence of all the 5 subtypes of SSTR (including 2A and 2B SSTR isoforms) in some surgically treated human thyroid tumors by means of immunohistochemistry and real-time PCR method and to correlate the results obtained with both techniques. SSTR 1 protein was expressed in 88.8% of investigated cases, SSTR 2A and 2B both in 44.4%, SSTR 3 in 55.5%, SSTR 4 in 11.2% and SSTR 5 in 33.3%. SSTR 1 is the dominant form in the thyroid gland tumor and hyperplasia. We found positive confirmation of both methods in 88.8% for SSTR 1, 2A, 3 subtypes, in 22.2% for SSTR 4 and in 100% for SSTR 5. It suggests that somatostatin multiligand analogs or selective SSTR 1 agonists may be used in thyroid tumors treatment.

## Background

Somatostatin (SST) as well as its synthetic analogs are engaged in regulation of hormones secretion in different ways and this action depends on specific receptors expression which are present on the target cells. Five subtypes of the SST receptor have been identified so far, i.e. SSTR 1–5. SSTR 2 is present in two splicing variants (2A and 2B) [1,2]. All of them belong to a group of 7 transmembrane domains linked with the G protein [3] and are encoded by 5 genes which are present on separate chromosomes [4,5]. In the cell the SST receptors can be found on the cell membrane or in the cytoplasm. Particular SSTR subtypes can occur alone or be grouped together in the same cell. SSTR are present both in normal and in tumor tissues which enables their response to applied SST analogs [6]. Human endocrine tumors often express the somatostatin receptors SSTR 1–5 with different intensity. It has been widely investigated their distribution in pituitary adenomas, brain tumors, adrenal tumors and neuroendocrine tumors in gastrointestinal tract (NET). Some of studies also concern the expression of SSTRs in thyroid tumors but they are mainly limited to parafollicular C cells – derived medullary thyroid carcinomas (MTC) [7-9]. Results of SSTR 1–5 detection in other thyroid pathologies like follicular adenomas and papillary cancers are still scarce and often controversial [10-13] depending of investigation method used (see Table 1).

**Table 1:** Expression of somatostatin receptors subtypes in some thyroid lesions – the previous studies

**Lesion type**	**SSTR detected**	**Material**	**Methods**	**Authors**
Follicular carcinoma	SSTR 1, 2A, 2B, 4, 5 -100%, SSTR 3-negative.	Formalin fixed paraffin embedded sections.	IHC	[12]
	SSTR 1, 3 -moderate, SSTR 2-faint, SSTR 5-strong, SSTR 4-negative.	Thyroid cancer cell line monolayer and xenograft (MRO-87, WRO-82).	Semiquantitative RT-PCR with ethidium bromide.	[10]

Papillary carcinoma	SSTR 1, 2B, 3, 4 -83%; SSTR 2A-66%, SSTR 5–100%.	Formalin fixed paraffin embedded sections.	IHC	[12]
	SSTR 1, 3, 5 -moderate, SSTR 2 – faintly detectable, SSTR 4 – negative.	Thyroid cancer cell line monolayer and xenograft (NPA 87, KAT-10).	Semiquantitative RT-PCR with ethidium bromide.	[10]
	SSTR 2 -87%.	Paraffin embedded sections.	IHC	[13]
	SSTR 2–68%, SSTR 5–50%, SSTR3 -31%, SSTR 1, 4 – not found.	Frozen tissue.	RT-PCR	[13]
	SSTR 1, 3, 4, 5-more than twice the background, SSTR 2 -no signal.	Frozen biopsied tumor tissue.	Northern blot analysis.	[21]

Multinodular goiter (MNG)	SSTR 2A, 3, 4 -82%, SSTR 2B, 5–94%, SSTR 1–88%.	Formalin fixed paraffin embedded sections.	IHC	[12]

Majority of studies applied the molecular biology methods of SSTRs investigation. These include the reverse – transcriptase PCR (RT-PCR), in situ hybridization (ISH), real-time PCR (RT-PCR) and Northern-blotting which are searching the expression of receptors at the level of mRNA. Apart from that, molecular biology methods are often burdened with some limitations like the necessity to use frozen tissue from surgical samples, very expensive reagents to perform the molecular reaction and sometimes gives false positive results. SSTRs investigation by immunohistochemical methods (IHC) is searching the expression of receptors at the level of receptor protein and gives us insight into receptor's cellular localization. The immunohistochemical technique can be also performed on paraffin embedded tissue specimens obtained from surgically removed tumors which are routinely used for histopathological examinations and seems to be the best *ex vivo *in vitro method in detecting cellular distribution of SST receptors [14]. In our previous study we proved high efficacy of immunohistochemical detection of SSTR expression in human pituitary adenomas, adrenal gland tumors and neuroendocrine tumors using antibodies specific for a given receptor subtype [15-17]. This method should be considered supplementary or even equally effective as methods of molecular biology or in vivo receptor radiodiagnostic imaging.

The aim of this study was to report the presence of all the 5 subtypes of SSTR (including 2A and 2B SSTR isoforms) in some surgically treated human thyroid tumors by means of immunohistochemistry and real-time PCR method and to correlate the results obtained with both techniques.

## Methods

In this study 9 samples obtained during surgically treated thyroid diseases were assessed. The material was obtained from 7 women and 2 men aged between 32 and 79 years (mean age 56 years). Four samples (nr 1–4) were diagnosed histopathologically as thyroid cancers, five samples (nr 6–10) concerned benign lesions of the thyroid gland (Table 2). This project received the approval of the Ethics Committee of Medical University of Łódź nr: RNN/97/06/KE dated 16.05.2006.

**Table 2:** Expression of somatostatin receptor subtypes in some thyroid gland diseases determined by IHC

**No**	**No of patient**	**Histopathological determination**	**SSTR – 1**	**SSTR – 2A**	**SSTR – 2B**	**SSTR – 3**	**SSTR – 4**	**SSTR – 5**
**P-1**.	**338542** F-56 years old	**Papillary carcinoma**	+++ mem, part. cytopl	+++ mem, part. cytopl	+ mem, pale cytopl	+/++ cytopl	negative + single cells	++ cytopl

**P-2**.	**333508** F-37 years old	**Papillary carcinoma**	+++ cytopl	++ mem/cytopl	++/+++ cytopl	++/+++ cytopl	-/+++ cytopl !!!	-/+++ cytopl

**P**-**3**.	**334708** F-79 years old	**Poorly differentiated carcinoma**	++ mem/cytopl	+ mem/cytopl	+ mem/cytopl	++ cytopl	negative	+/-/+ cytopl

**P**-**4**.	**335385** M-69 years old	**Poorly differentiated carcinoma**	+++ mem/cytopl	+/-;	+ cytopl	+/-	negative	+/-/++ cytopl

**P**-**6**.	**332314** F-45 years old	**Lymphatic goiter**	negative	negative	negative	negative	negative	+++ cytopl (in the single follic. struct.)

**P**-**7**.	**332142** F-32 years old	**Nodular hyperplasia of thyroid gland**	++ cytopl	+ cytopl	++/+++ cytopl	+++ cytopl	negative	+++ cytopl

**P**-**8**.	**332567** M-73 years old	**Colloido-nodular goiter. Follicular adenoma**	++/+++ cytopl	++ cytopl	+ cytopl	++/+++ cytopl	negative	+/++ cytopl

**P**-**9**.	**332131** F-67 years old	**Thyroid goiter with signs of focal hyperactivity**	++ cytopl	+/- cytopl	++ cytopl	+ cytopl	negative	+ cytopl

**P**-**10**.	**332205** F-44 years old	**Nodular hyperplasia of thyroid gland**	+++ cytopl	++ cytopl	++ cytopl	+++ cytopl	negative	+ cytopl

### Immunohistochemistry

Bouin's fixed, dehydrated and paraffin embedded 8-μm sections were immunostained using commercially available rabbit polyclonal antisera raised against carboxyl-terminal fragments of specific human somatostatin receptor subtypes (GRAMSCH Laboratories, Schwabhausen, Germany): SSTR 1 (named SS-840 antibody, corresponding to amino acid sequence 377–391 of the receptor's peptide chain), SSTR 2A (SS-800, specific for 355–369 sequence), SSTR 2B (SS-860, specific for 342–356 sequence), SSTR 3 (SS-850, specific for 381–395 sequence), SSTR 4 (SS-880, specific for 374–388 sequence) and SSTR 5 (SS-890, specific for 350–364 sequence). The immunohistochemical procedures were performed as previously described [18]. The working dilution of antibodies was 1: 1000 (diluted in 0.05 M TRIS buffer, pH 7.6 containing 2% goat serum). Following overnight incubation in 4°C in humidified chamber with primary antibodies, the cells were treated with anti-rabbit IgG biotinylated goat antibody (1:800, DAKO, Denmark) and streptavidin complex (Strept ABC/HRP, DAKO, Denmark). The immunoreaction was visualized with 3,3'-diaminobenzidine (DAB, DAKO, Denmark) solution. For negative controls the primary antibody was omitted and the normal goat serum was used.

The immunoreactive intensity for specific receptor proteins was scored semiquantitatively using a descriptive scale as follows: strong staining (+++), moderate staining (++), weak staining (+) and pale staining (+/-). Subcellular distribution pattern of SSTR subtypes – membranous or cytoplasmic was also determined.

### Real-Time PCR

Total RNA was isolated from 1 g of frozen tissue using Magna Pure Compact RNA Isolation Kit Cat. No 04 802 993 001 (Roche Molecular Biochemicals) from nine patients. Total RNA was DNase treated using DNase I (RNase free) reagent (Ambion Cat. no AM2222). First-strand cDNAs were synthesized from equal amounts of total RNA (0,5 μg/reaction) using oligo(dT) and iScript cDNA Synthesis Kit (Bio-Rad1Cat. No70-8890) according to the manufacturer's instruction. All primers were purchased in Oligo PAN Warszawa (Table 3) [19,20]. RT-PCR was performed using pairs of primers in reaction amplifying across a gradient of annealing temperatures to identify optimal reaction conditions for Real-Time PCR. Real – Time PCR was performed using an iCycler iQ System (Bio-Rad Cat No 170-8701, 170-9750). The rate of accumulation of amplified DNA was measured by continuous monitoring of SYBR Green I fluorescence. Melt Curve of the reaction products were in each experiment generated.

**Table 3:** Primers sequences applied for quantitative Real-Time PCR

Name	Sequence	Size of PCR product, bp	GenBank accession no.
**SSTR 2A (forward)** **SSTR 2A (reverse)**	**5'ATGCCAAGATGAAGACCATCAC 3'** **5'TGAACTGATTGATGCCATCCA 3'**	171 [19]	NM 001050

**SSTR 3 (forward)** **SSTR 3 (reverse)**	**5'CTGGGTAACTCGCTTGGTCATCTA 3'** **5'AGCGCCAGGTTGAGGATGTA 3'**	86 [19]	NM 001051

**SSTR 1 (forward)** **SSTR 1 (reverse)**	**5'TATCTGCCTGTGCTACGTGC 3**' **5'GATGACCGACAGCTGACTCA 3**'	217 [20]	NM 001049

**SSTR 4 (forward)** **SSTR 4 (reverse)**	**5'ATCTTCGCAGACACCAGACC 3'** **5'ATCAAGGCTGGTCACGACGA 3'**	321 [19]	NM 001052

**SSTR 5 (forward)** **SSTR 5 (reverse)**	**5'GTGACAACAGGACGCTGGT 3'** **5'TGGTGACGGTCTTCATCTTG 3'**	156 [20]	NM 001053

**HPRT (forward**) **HPRT (reverse)**	**5'TGCTTTCCTTGGTCAGGCAGTAT 3'** **5'TCAAATCCAACAAAGTCTGGCTTATATC3**'	109 [19]	NM 000194

Specifically, quantitative real-time PCR on the iCycler iQ was performed in triplicate on 1 μl of template cDNA per 25 μl reaction. iQ supermix reactions consisted of iQ SYBR Green Supermix (Bio-Rad1Cat. No70-8882) at a final concentration 1×, 10 nM fluorescein calibration dye, SYBR Green I, 500 nM of each primer. Reactions were amplified in a 96-well thin-wall PCR plate (Bio-Rad Cat. No 223-9441) using the following PCR parameters: 95°C for 3 min followed by 50 cycles of 95°C for 10 sec, 60°C for 30 sec, and 72°C for 30 sec. Melt-curve analysis was performed immediately following amplification by increasing the temperature in 0,4°C increments starting at 60°C for 85 cycles of 10 sec each. The presence of a single PCR product was verified by the presence of a single melting temperature peak.

Real-time RT-PCR reactions for detection of the endogenous control gene (HPRT) were always run in parallel in each experimental run as a reference for accuracy of sample dilution.

For each experiment the 3-point standard curve was performed with commercial control RNA [Apllied Biosystems Control Total RNA Human 4307281 (100 μl conc. 50 ng/μl) – dilution 1×, 10× and 100×)].

## Results

### Immunohistochemistry

**Figure 1:**
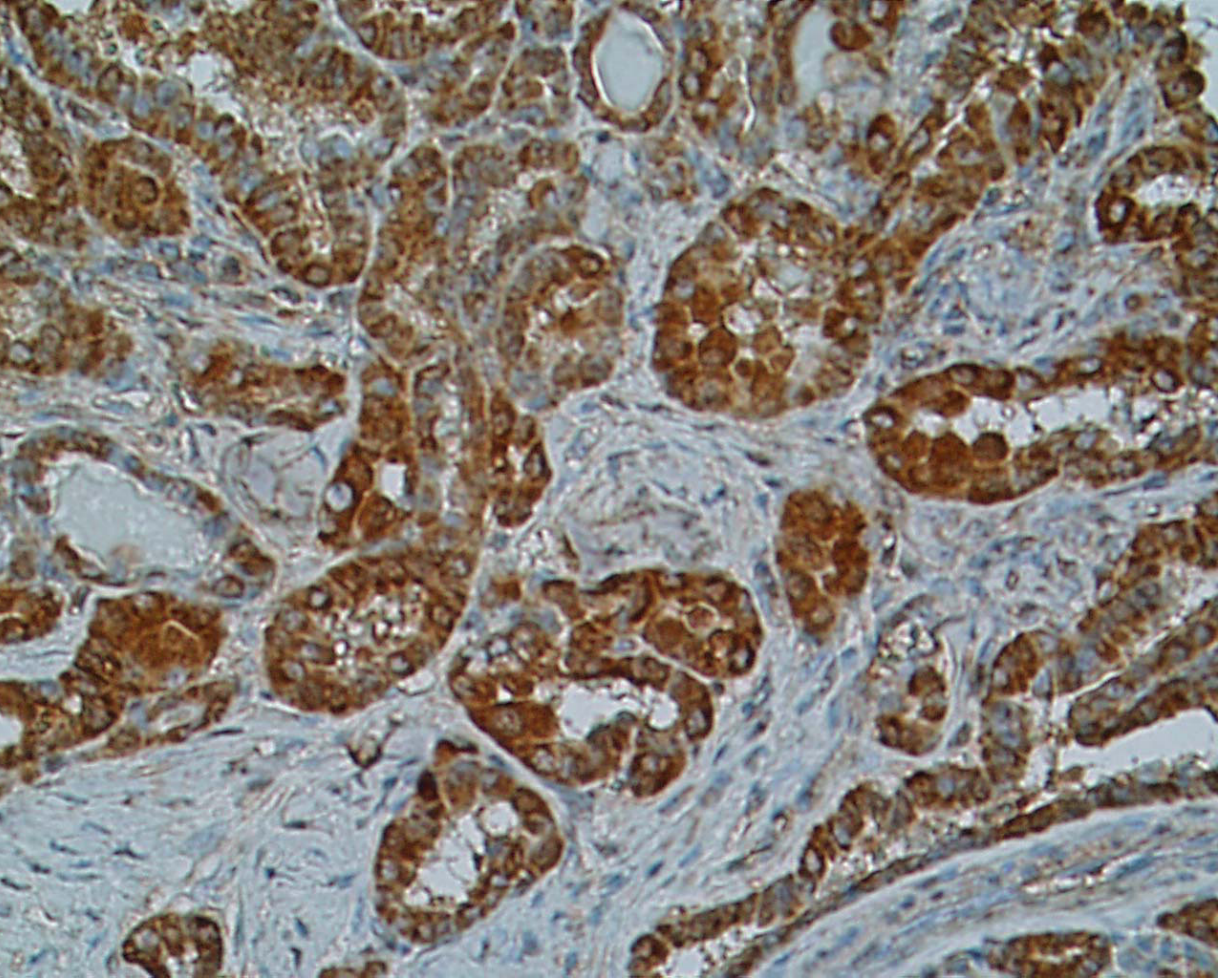
**Immunostaining of SSTR 1 in papillary carcinoma, patient nr 2 (333508) (× 200)**. Strong intensity of staining distributed in the cytoplasm.

**Figure 2:**
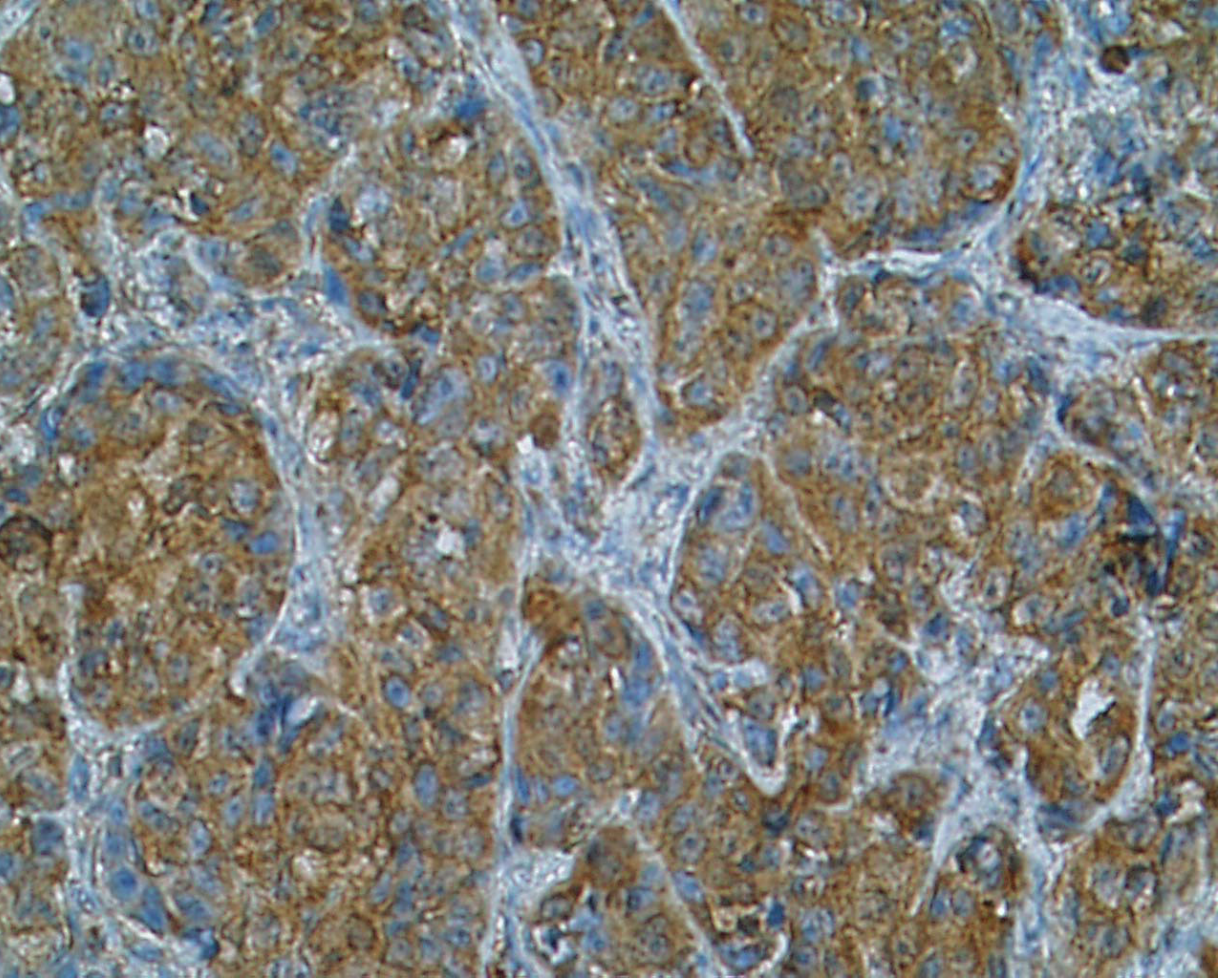
**Immunostaining of SSTR 1 in poorly differentiated carcinoma, patient nr 3 (334708) (× 200)**. Moderate staining with both cytoplasmic and membranous distribution.

**Figure 3:**
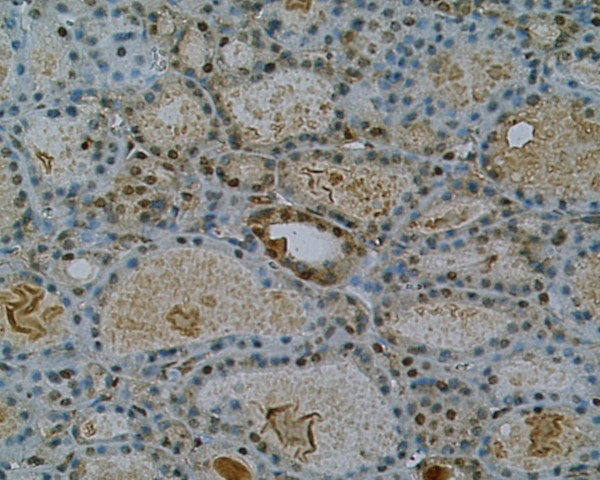
**Weak immunostaining of SSTR 3 in non-malignant goiter, patient nr 9 (332131) (× 200)**. Cytoplasmic distribution with weak intensity of staining.

**Figure 4:**
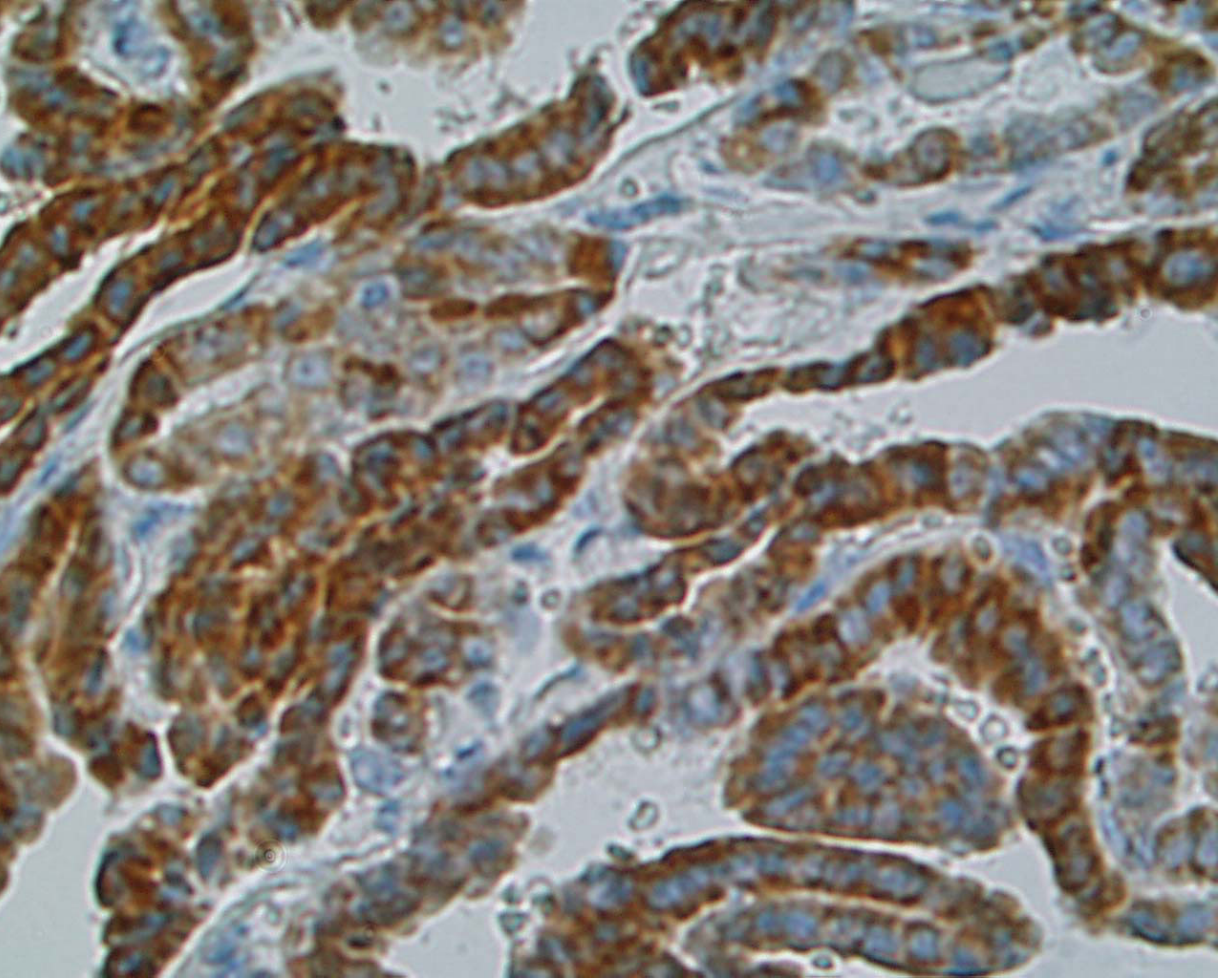
**Immunostaining of SSTR 4 in papillary carcinoma, patient nr 2 (333508) (× 400)**. Cytoplasmic distribution of this receptor subtype fluctuates from pale to strong.

Immunoreactivity of investigated thyroid tissues was estimate in pathologically altered cells of thyroid cancers and benign lesions of the thyroid gland. The area of normal surrounding tissue was immunonegative in all investigated cases. Multiple SSTR subtypes were found to coexist in each case of investigated tissue (Table 2). Most of the specimens showed mixed distribution pattern of the receptors – cytoplasmic and membranous. Strong and moderate staining of **SSTR 1 **which was distributed both in the cytoplasm and the membranes or only in the cytoplasm was shown in 8 (88,8%) specimens (Fig. 1),(Fig. 2). **SSTR 2A **was also characterized by mixed distribution – cytoplasmic and membranous. Strong and moderate staining was demonstrated in 4 cases (44,4%) and in the remaining 4 cases (44,4%) distribution of this SSTR subtype was fluctuated from weak to pale. In 4 specimens (44,4%) strong to moderate staining of **SSTR 2B **was observed with both membranous distribution and in the area of the cell cytoplasm. In the other 4 cases the staining intensity was weak or pale. Staining for **SSTR 3 **was characterized by cytoplasmic distribution with various intensity. In 5 (55,5%) out of 9 examined specimens SSTR 3 was found with strong or moderate intensity in the cell cytoplasm and the remaining 3 cases (33,3%) showed weak to pale intensity of staining (Fig. 3). There was no immunostaining of the SSTR 1–3 subtypes in patient nr 6 with lymphatic goiter. In this case the immunoreactivity of SSTR 1, 2A, 2B and 3 was negative in the thyroid follicular epithelium but pale to strong staining was observed in the lymphatic follicles. No presence of **SSTR 4 **was found in the 8 (88,8%) of investigated cases with exception of 1 (11,2%) specimen showing cytoplasmic distribution of this receptor subtype fluctuating from pale to strong (patient nr 2) (Fig. 4). Similarly to SSTR 3, **SSTR 5 **was also characterized by exclusively cytoplasmic distribution with strong and moderate intensity in 3 samples (33,3%). Another 5 (55,5%) cases demonstrated pale to moderate staining. In 1 (11,2%) specimen the intensity of staining was from pale to strong (patient nr 2).

### Real-Time PCR

**Figure 5:**
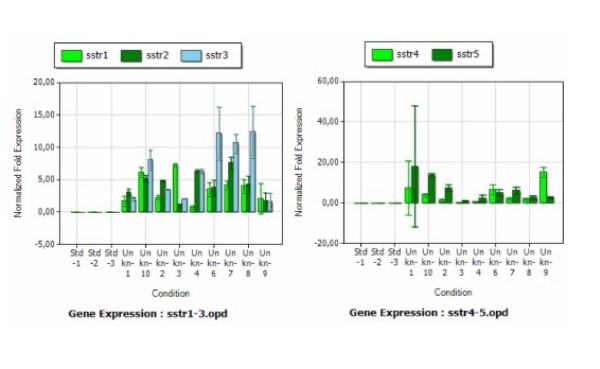
**Somatostatin 1, 2A, 3, 4, 5 receptor subtypes gene expression determined by RT-PCR method**. mRNAs for SST receptors subtypes were calculated relative to the amount of HPRT control gene and given in arbitrary units. Std-standard 1,2,3 of endogenous control gene. Unkn – expression of SSTR mRNA in patients (1–4), (6–10). sstr1, 2, 3, 4, 5 = SSTR 1, 2A, 3, 4, 5 mRNA.

**Figure 6:**
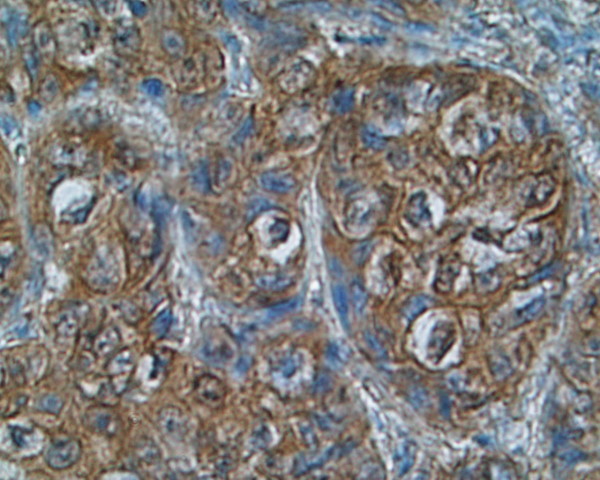
**Immunohistochemical staining of SSTR 2A in poorly differentiated carcinoma, patient nr 3 (334708) (× 400)**. Weak intensity of SSTR 2A protein staining with mixed cytoplasmic and membranous distribution.

We consolidated result for endogenous control gene of hypoxanthine-guanine phosphoribosyltransferase (HPRT) as negative (score: 0,0) and according to this all remaining mRNAs were calculated relative to the amount of HPRT and given in arbitrary units. The diagram presented below show the mean results of 3 experiments for each primer (Fig. 5). SSTR 1 mRNA was expressed in all isolated tissues with different intensity. In 7 from 9 cases (77,7%) it is correlated with immunohistochemical investigation of SSTR 1 where intensity of staining was from moderate to strong. In 1 patient (nr 4) SSTR 1 mRNA was only slightly elevated while expression of SSTR 1 protein in IHC was strong. The inverse situation we observed in patient nr 6 where SSTR 1 mRNA was elevated while protein expression was negative. SSTR 2A mRNA was elevated in 6 patients (66,6%) thus a good correlation of RT-PCR and IHC results was observed in patients: 1,2,7,8,9,10. Patient nr 3 with poorly differentiated carcinoma had expression of SSTR 2A protein with weak intensity (Fig. 6) and this result was also in agreement with low score of RT-PCR. It gives summary 77,7% of good correlation. The different situation appeared in patients nr 4 and 6 (22,2%) were pale or negative immunostaining of SSTR 2A and 2B was not correlated with high score of SSTR 2A gene expression. The same results in these patients (nr 4, 6) (22,2%) we observed in case of SSTR 3 subtype. Pale or negative immunohistochemical score was not correlated with high score of SSTR 3 gene expression. Immunostaining of SSTR 3 was in agreement with RT-PCR method in 7 cases (patients: 1,2,3,7,8,9,10) (77,7%) were strong and moderate staining corroborates with the presence of high and elevated score of SSTR 3 mRNA. Only two negative results of SSTR 4 mRNA estimation (22,2%) were good correlated with negative immunohistochemical staining (patients: 3,4). No immunostaining of SSTR 4 was observed in patients: 1,6,7,8,9,10 but a slightly or elevated gene expression was observed in them. In 1 sample (patient nr 2) exceptional fluctuated distribution of SSTR 4 from pale to strong was correlated with faintly detectable gene expression. In all 9 samples (100%) high or low score of SSTR 5 mRNA expression was well correlated with strong to pale IHC staining.

## Discussion

**Figure 7:**
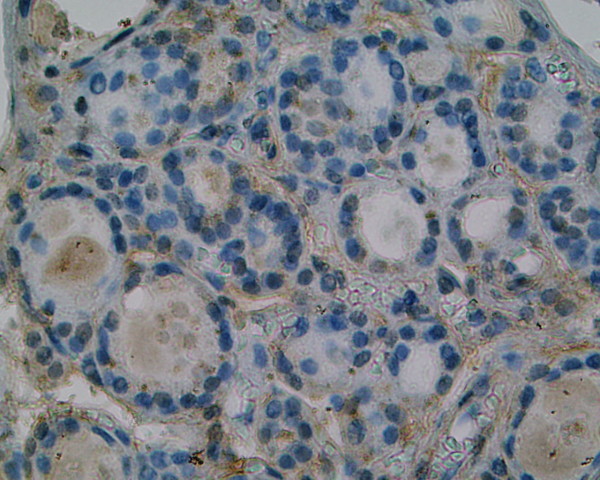
**Pale immunostaining of SSTR 2A in non-malignant goiter, patient nr 9 (332131) (× 400)**. Pale cytoplasmic expression of this receptor subtype.

We demonstrated in our investigation that somatostatin subtype receptors are widely distributed in human thyroid diseases with different intensity and cellular localization. It is consists with earlier reports of England et al.[12] who find positive immunostaining in number of investigated tissues in relation to type of thyroid pathology. The cited authors have found that for 6 investigated cases with papillary carcinomas 5 patients had SSTR 1, 2B, 3 and 4 receptor subtype (83%). In all of them SSTR 5 was observed (100%) and SSTR 2A was expressed in 4 cases (66%). In multinodular goiters SSTR 2A, 3 and 4 were expressed in 82% cases, SSTR 2B, 5 in 94% and SSTR 1 was expressed in 88%. In our IHC study in 88,8% of investigated cases we find SSTR 1 with strong and moderate immunostaining. In contrast, the expression of SSTR 2A and 2B was found in 44,4%, SSTR 3 in 55,5%, SSTR 4 in 11,2% and SSTR 5 in 33,3% of cases. Our results are conflicting with England et al. in the scope of SSTR 4, where we did not detect SSTR 4 at the protein level in multinodular goiter (MNG) cases using the immunohistochemical method. Maybe other type of specific anti-SSTR 4 antibody was used in both experiences. Thus our results show that SSTR 1 but not other subtypes is the dominant form in the thyroid gland tumors and hyperplasias. The molecular biology method results prove the predominance of SSTR 1, 2A and 3 mRNAs. These data suggest that despite of the conventional somatostatin analogs: octreotide and lanreotide, multiligand analogs such as SOM 230 or KE 108 or selective ligands of SSTR 1 may be useful in thyroid tumors treatment. Our material is to scarce to estimate the possible differences between benign and malignant thyroid lesions, however, poorly differentiated cancers characterize by low expression of SSTR 2 (Fig. 6). It is worth interesting that in cancer tissue the expression of SSTR 1, SSTR 2A, SSTR 2B may be membrane or cytoplasmic (Fig. 1), (Fig. 2), (Fig 6), whereas in non-cancer tissue the expression of these receptors was only cytoplasmatic (Fig. 7). This observation may suggest the higher responsiveness of SSTR in highly differentiated malignant tumors than in benign lesions. In the Druckenthaner's experiences [13] SSTR 2 immunohistochemistry was positive in 87% of the samples with thyroid tumors. SSTR 2 mRNA expression well correlated with immunohistochemical staining pattern in 13/16 samples (81%). In this study the SSTR 2 mRNA was the predominantly expressed on thyroid tumors (68%), SSTR 5 and SSTR 3 mRNAs were less predominant (50% and 31% respectively). No SSTR 3 and SSTR 4 mRNAs was found in any specimens. Also Forssell-Aronsson's group [21] reported that in surgical specimens SSTR 1, 3, 4, 5 mRNAs were frequent expressed but SSTR 2 mRNA was not detected. Similar results were observed earlier by Ain [10] but in the thyroid carcinoma cell lines not in surgically isolated tissues. Most thyroid cancer cell line monolayers and xenografts expressed SSTR 3 and SSTR 5 mRNAs whereas SSTR 2 mRNA was only faintly detectable. In our experiences we found positive confirmation of both methods in 77,7% for SSTR 1, 2A, 3 subtypes, in 22,2% for SSTR 4 and in 100% for SSTR 5. The lack of good correlation between receptor protein expression (positive immunostaining) and receptor gene expression (negative by RT-PCR and vice versa) in our study needs explanations by future examinations. Several causes should be taken into consideration, e.g. the expression of SSTR mRNA in tissue elements other than thyroid epithelial cells, like vascular endothelia, parafollicular cells and lymphoid cells which can lead to the false positive results of RT-PCR examination or the lower sensitivity of IHC which in turn can produce the false negative results. Lastly, the expression on the mRNA and protein levels should not to be always synchronized.

## Conclusion

We proved that somatostatin receptor subtypes are frequently expressed in pathologically altered thyrocytes, in contrast to normal thyroid follicular epithelium. SSTR 1 protein was expressed in 77,7% of investigated cases, SSTR 2A and 2B both in 44,4%, SSTR 3 in 55,5%, SSTR 4 in 11,2% and SSTR 5 in 33,3%. SSTR 1 is the dominant form in the thyroid gland tumor and hyperplasia. A good correlation with SSTR 1 receptor gene expression was observed in 77,7% of investigated cases. Immunohistochemical estimation of SSTR 2A and 3 was agree with RT-PCR method in 77,7%, only 22,2% of results of SSTR 4 mRNA estimation were correlated with immunohistochemical staining and SSTR 5 mRNA was good correlated with IHC staining in 100% tissues. It suggests that somatostatin multiligand analogs or selective SSTR 1 agonists may represent a further useful approach for the thyroid tumors treatment. The expression of somatostatin receptor subtypes in thyroid tumors needs father studies concerning greater and more differentiated group of patients.

## Abbreviations

mem: membranous localization; cytopl: cytoplasmatic localization; strong staining (+++), moderate staining (++), weak staining (+) and pale staining (+/-); SST: somatostatin; SSTR: somatostatin receptor subtype; RT-PCR: real time polimerase chain reaction; RT-PCR: reverse – transcriptase polimerase chain reaction; ISH: in situ hybridisation; IHC: immunohistochemistry; NET: neuroendocrine tumors in gastrointestinal tract; MTC: medullary thyroid carcinoma; MNG: multinodular goiter; mRNA: messenger ribonucleic acid; HPRT: hypoxanthine-guanine phosphoribosyltransferase control gene.

## Competing interests

The authors declare that they have no competing interests.

## Authors' contributions

HP carried out the immunohistochemistry studies and drafted the manuscript. TS obtained thyroid tissues during surgery. RK established histopathological diagnosis. EB carried out the molecular biology studies. MP supervised the experiences, helped to draft and revised the manuscript and provided general support. All authors read and approved the final manuscript.
